# Supplementary figures and images for: The N-terminal domain of RfaH plays an active role in protein fold-switching
Source: PLoS Comput Biol. 2021 Sep 3;17(9):e1008882. doi: 10.1371/journal.pcbi.1008882 (PMC8454952; doi:10.1371/journal.pcbi.1008882)

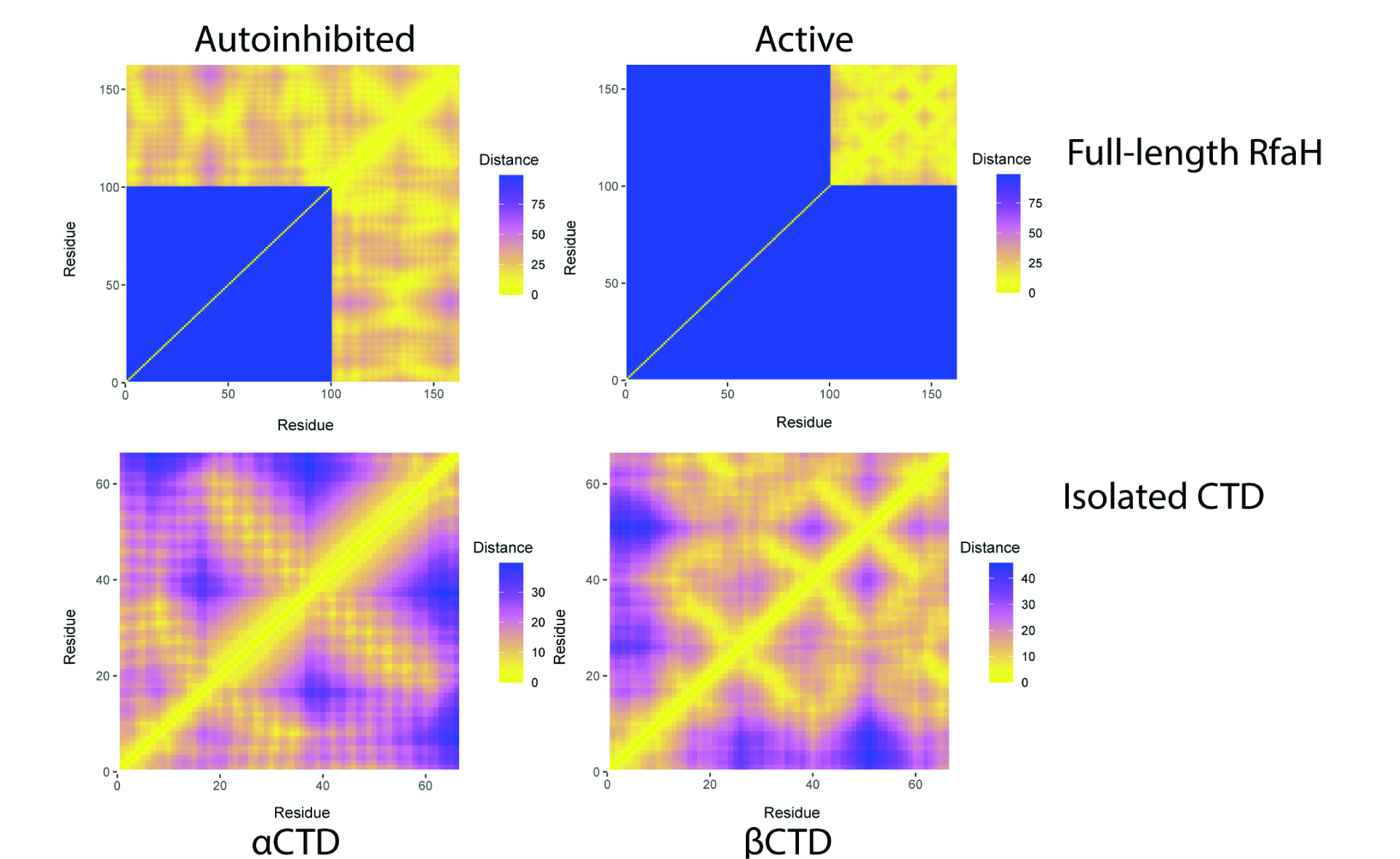

Supplement: S1 Fig — Cα residue-residue distance matrices for full-length RfaH and its isolated CTD. The matrices grow along the diagonal, which represents the same residue distance, in this case set to 0. Along this diagonal, contacts are formed in a 1–4 residue pattern for α-helices, antiparallel and parallel lines indicating β-strands. The blue blocks indicate regions of high distance (99 Å), which were manually set in order to exclude them from the Qdiff calculation. (TIF) [file pcbi.1008882.s002.tif]

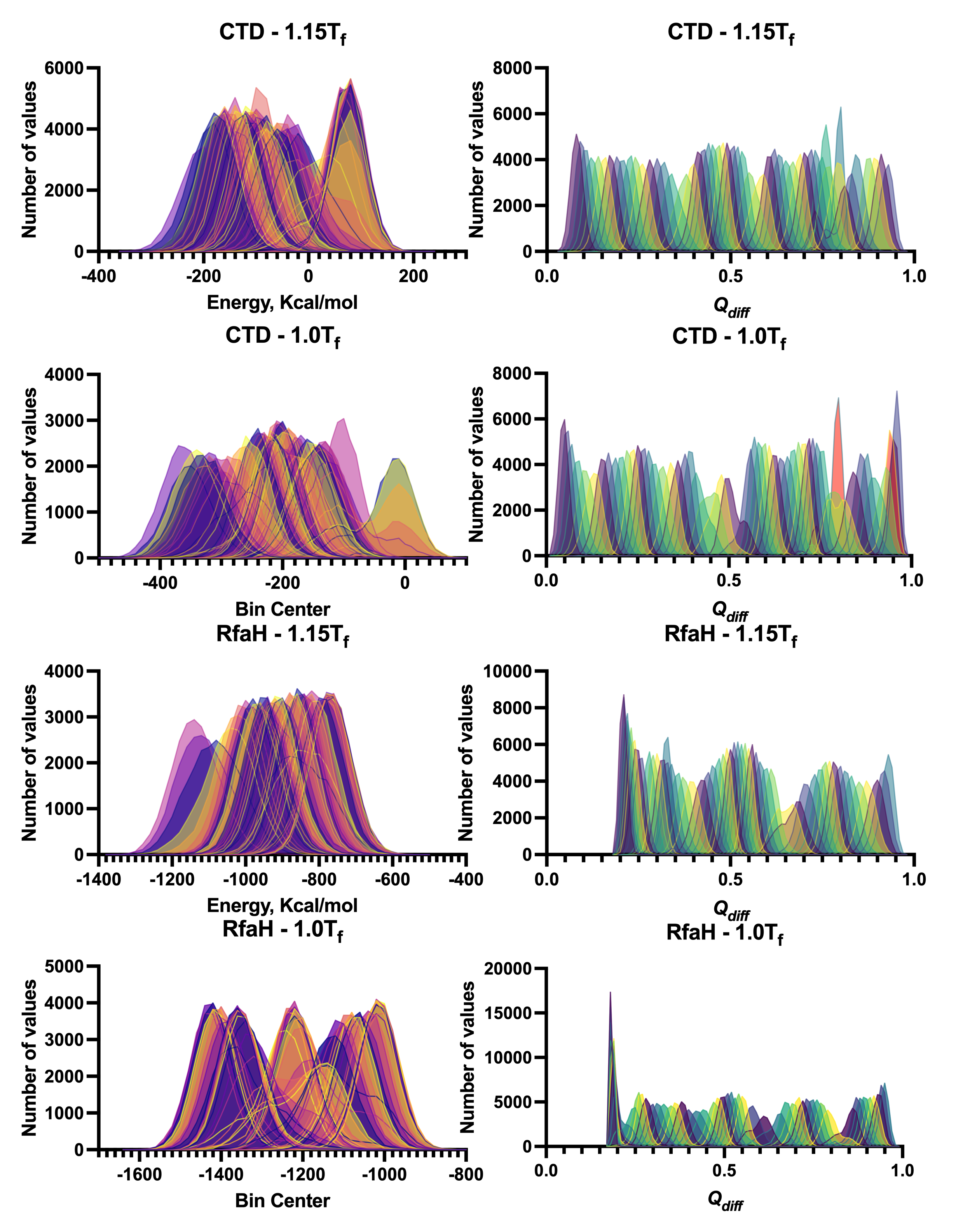

Supplement: S2 Fig — In these umbrella sampling simulations, 51 simulations in Qdiff steps of 0.02 were run, totaling 51 simulations per system per temperature. The histograms marked in red were not used for the WHAM analysis as the simulation got trapped in a misfolded configuration. RfaH reaches the α-folded autoinhibited state when Qdiff = 1 and the isolated CTD reaches the β-folded state when Qdiff = 1. Although not sufficient sampling was achieved for Qdiff ~ 0.00 for the full-length protein, the beta configuration was successfully sampled as it is observed in Fig 1B. (TIF) [file pcbi.1008882.s003.tif]

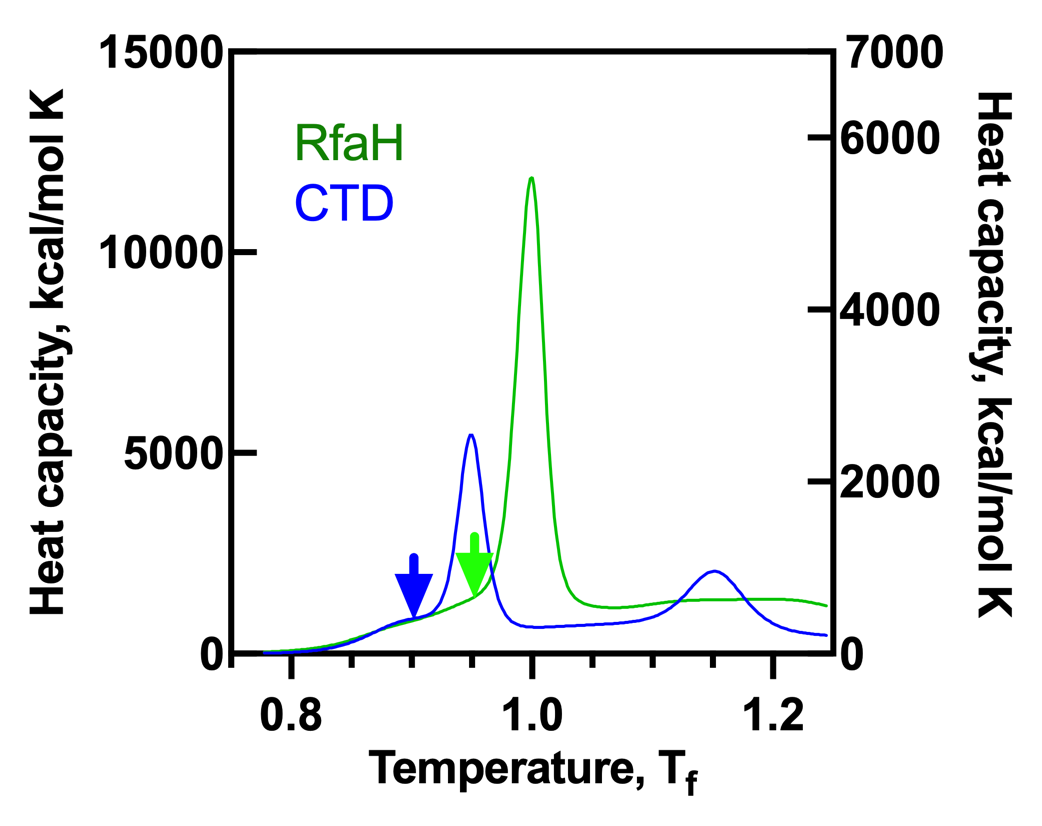

Supplement: S3 Fig — Heat capacity calculated from umbrella simulations on the full-length RfaH and the isolated CTD. The blue arrow indicates the temperature selected for presenting the free energy landscape of the isolated CTD in Fig 2A, and the blue arrow indicates the temperature selected for presenting the free energy landscape of the full-length RfaH in Fig 2B. The values on the left y-axis correspond to RfaH, whereas the values on the right y-axis correspond to the isolated CTD. (TIF) [file pcbi.1008882.s004.tif]

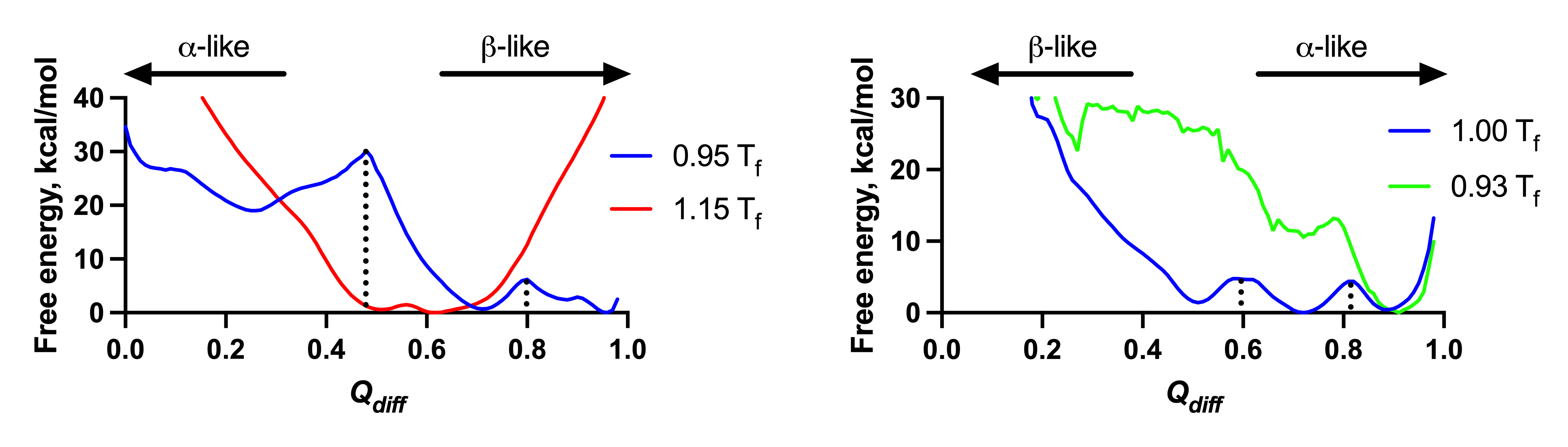

Supplement: S4 Fig — The free energy landscapes of isolated CTD (left) or full-length protein (right) were projected onto the Qdiff reaction coordinate alone, which describes the transition between α-folded and β-folded CTD. (TIF) [file pcbi.1008882.s005.tif]

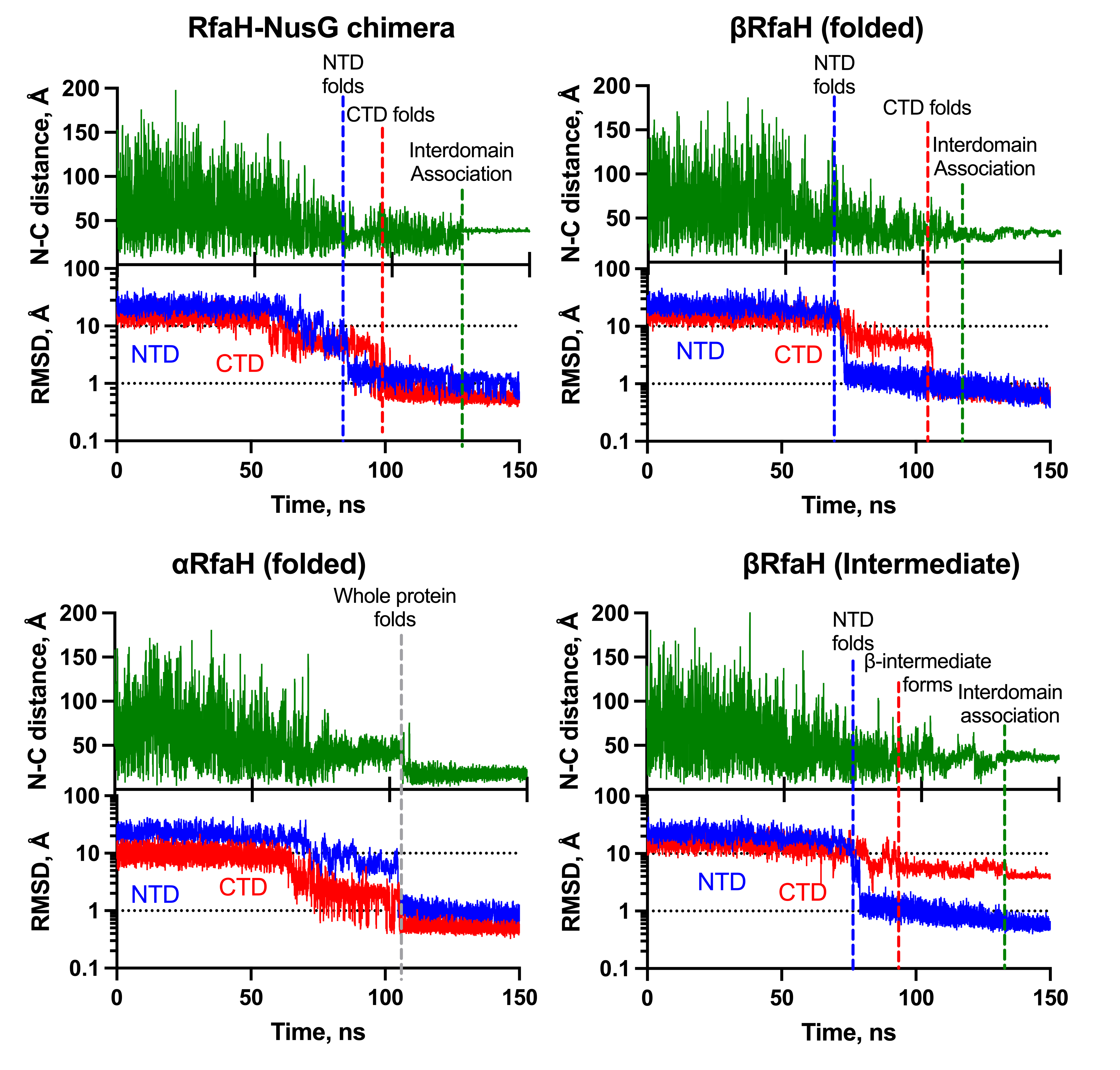

Supplement: S5 Fig — The N-C distance shown in green is a measure of how close or separated are the proteins. At low temperatures they tend to agglutinate as a way to minimize the energy, particularly of the exposed NTD hydrophobic patch, which has many residues whose burial energy remains unsatisfied otherwise. (TIF) [file pcbi.1008882.s006.tif]

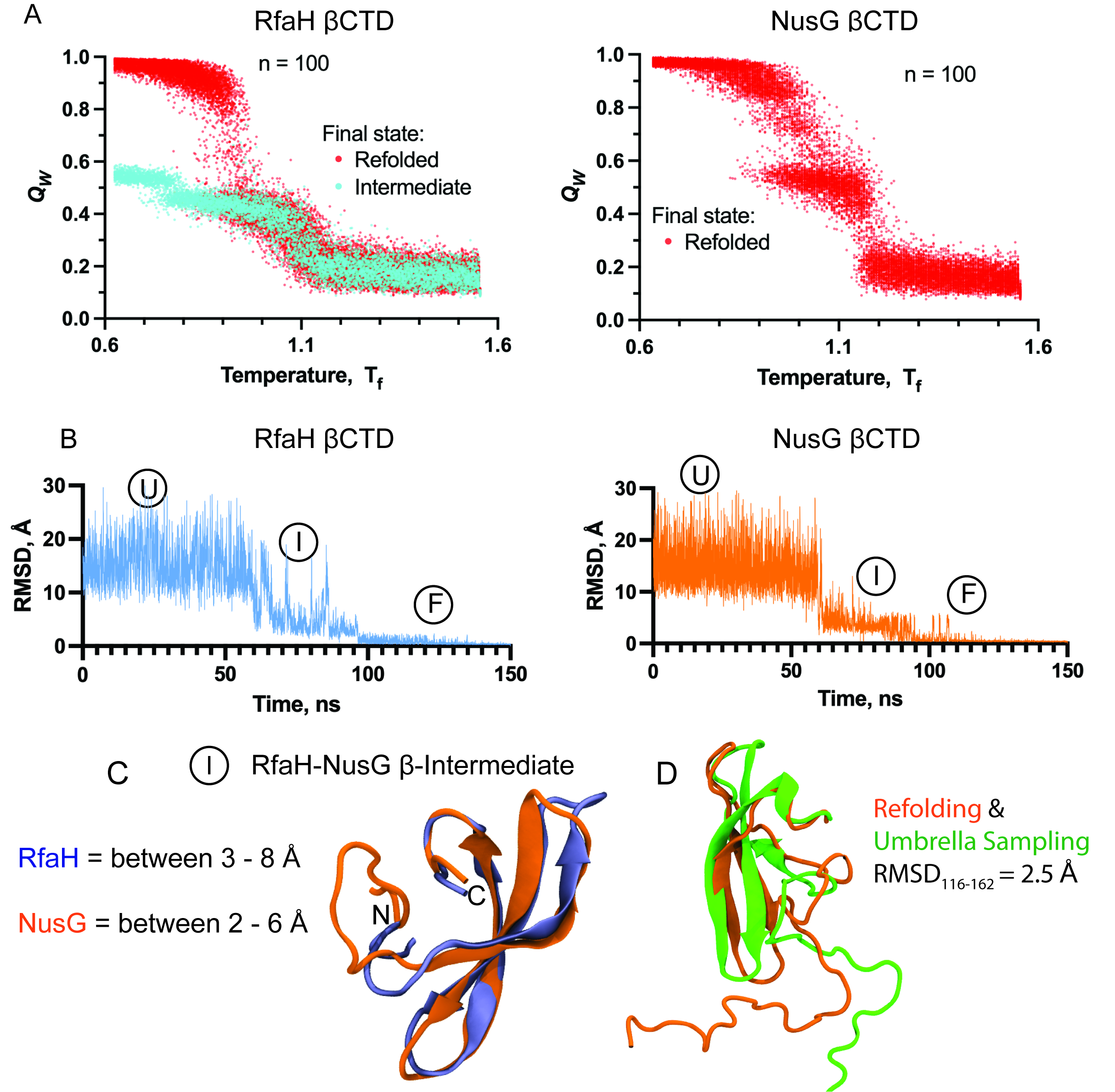

Supplement: S6 Fig — (A) Annealing plots of RfaH CTD and NusG CTD. Each point was taken every 2,000 steps of 3·107 step trajectories that ramped down from 1.6 Tf to 0.6 Tf. For both RfaH and NusG, an intermediate is observed at 0.4 ≤ QW ≤ 0.6. (B) Comparison of refolding traces and intermediate structures of RfaH CTD and NusG CTD. The folding states of both traces was visually inspected. For each trace, the unfolded state is denoted as U, while the intermediate state is denoted as I and the folded state is denoted as F. (C) Structural alignment via STAMP of the intermediate states observed for RfaH and NusG and the RMSD to the folded state for RfaH and NusG. (D) Structural alignment via STAMP of the β-intermediate state observed for RfaH CTD in umbrella sampling and refolding simulations. (TIF) [file pcbi.1008882.s007.tif]

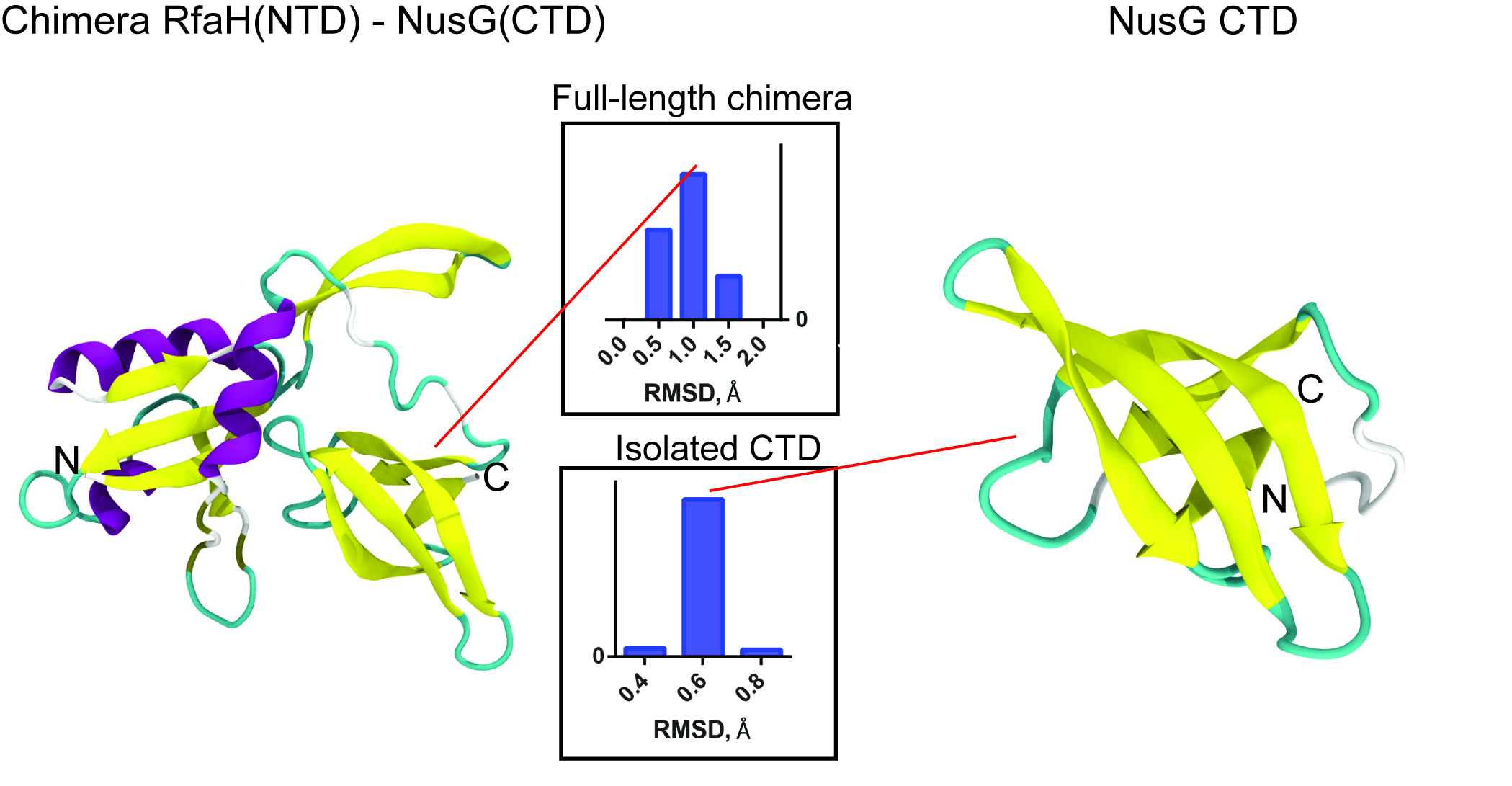

Supplement: S7 Fig — Representative final structures after NusG βCTD refolding in a RfaH NTD–NusG CTD chimera and in isolation. The histograms represent the RMSD distribution of the final structures. All simulations reached the β-folded state of NusG CTD. (TIF) [file pcbi.1008882.s008.tif]
