# Supplementary material for: The N-terminal domain of RfaH plays an active role in protein fold-switching
Source: PLoS Comput Biol. 2021 Sep 3;17(9):e1008882. doi: 10.1371/journal.pcbi.1008882 (PMC8454952; doi:10.1371/journal.pcbi.1008882)
Supplement: Attachment — Submitted filename: Response_Reviewers_rev1.docx [file pcbi.1008882.s009.docx]

**Responses and corrections to reviewer’s comments:**

Author’s comments and responses are shown in red.

**Reviewer #1:** RfaH is one of the most extreme examples of a fold-switching protein. The two-domain protein reversibly cycles between two states: in the closed state the C-terminal domain (CTD) folds as a-helical hairpin and binds to the N-terminal domain, rendering RfaH autoinhibited. Upon recruitment to an ops-paused TEC the domains dissociate and the CTD refolds to a NusG-like b-barrel. Although many details of its functional cycle have been deciphered and despite many bioinformatical studies, the molecular basis of CTD refolding is still largely unknown.

In this manuscript the authors use Associative Water-mediated Structure and Energy Model (AWSEM) molecular dynamics applying umbrella sampling and temperature refolding simulations in order to study the transformation of the CTD with a particular focus on how the NTD affects CTD folding. First they confirm the experimentally determined folding states of full-length RfaH and the isolated CTD exploring their conformation space, but, interestingly, their analysis reveals a β-intermediate, which consists of three β -strands and which may be a general folding intermediate in the folding pathway of the β -barrel. The authors show that this intermediate is stabilized by contacts to the NTD and they hypothesize that a destabilization of the β -barrel by the NTD is the first step when RfaH transforms from its activated to the autoinhibited state.

Overall, the manuscript is sound and reveals new aspects of the transformation of RfaH.

Comments

1. p. 5, l 9: A general explanation of the “umbrella sampling” should be added so that also non-bioinformaticians can read and understand this section.

We thank the reviewer for raising this issue. We added a brief paragraph detailing the basics of the computational experiment (p 7-8)

2. Fig. 1B: labeling of the points at which the energy landscapes are calculated should be colored according to the curves to facilitate understand; it is also not clear why the letters “C” and “R” are chosen so that arrows colored according to the curves should be sufficient

The figure was moved to supplementary data (Fig. S3), and the correction was performed.

Fig. 1C: The energy barrier between αRfaH and βRfaH should be marked

The umbrella sampling simulations we ran are too biased to the desired configurations (as the harmonic restraint was 1,500 kcal/mol), hence no equilibrium transition between both states was observed. Nevertheless, after observing that *Q_diff_* is a good reaction coordinate to discern between the configurations (please see Fig. 2), we calculated the 2D profiles for these transitions using only Qdiff as reaction coordinate, where the barriers can be seen (Fig. S4).

3. p.7, l 1-3: Contacts of the flexible linker have been ignored as no defined conformation has been reported, which is reasonable. Nevertheless, the linker might play a role during refolding (either to α or β or both states). This possibility should be included in the discussion.

We thank the reviewer for raising this issue. We corrected the description as the linker indeed was included in the contacts to be evaluated, although it forms only a few contacts compared to the CTD (please refer to Fig. S1 to verify this). In the umbrella sampling, the main force directing the exploration is one caused by biased contact rupture/formation. Therefore, the presence or absence of linker contacts is only relevant for the sampling and will not necessarily determine the shape of the resulting free energy surface. Based on this and on previous bibliography, we added a paragraph to the discussion that explores the effect of the linker in stabilizing any fold (p 20)

4. p. 7, l 19-20: The authors should describe the graphs of Fig. S2 and how exactly they relate to the energy landscapes in Fig. 1 (not only technically, i.e. that it’s the 1D projection, but in terms of minima etc). They should also describe how the β-intermediate has been identified/observed (minimum). As this is the first time that the β-intermediate has been identified, the Fig. S2 should be moved to the main manuscript.

Thanks for this suggestion. We added an additional free energy landscape in our new Fig. 2 to identify the β-intermediate and describe it in p 11. We also replaced Fig S2 by a projection of RfaH transformation along Qdiff as reaction coordinate, which is available in Fig S4.

5. p. 8, l 24-25: This kind of behavior is expected for a two-domain proteins the domains of which behave independently not for a two-domain protein in general -> rephrase

Thanks for the suggestion. The phrase was changed.

6. Fig. 2B and C: to clarify the states they should be labeled “correctly folded domains, correct interface” (B) and “correctly folded domains, incorrect interface” (C); the color code should be explained

Corrected as indicated by the reviewer in Fig. 3.

7. p. 9, l 10: The refolding simulations of full length RfaH not only recapitulate the structure of RfaH (Ref 8), but also the experimental unfolding/refolding experiments (ref 24)

The reviewer is correct, as the spontaneous refolding to the alpha-state has been experimentally determined in such reference. This is now added in p 15, thanks for the suggestion

8. p. 9, l 16-17. It is not clear why solely the NTD-side of the domain interface is responsible for the correct orientation/binding of the CTD; such a conclusion requires a detailed analysis of the contacts and is only valid if specific sidechains of the NTD contact only backbone atoms from the CTD; if also CTD sidechains are involved the specificity relies on both domains

We thank the reviewer’s concern. On the new Fig S5 is observed (as in all trajectories analyzed) that the αCTD becomes stable only simultaneously or after the NTD folds. This finding suggests that the NTD is essential to stabilize the α-hairpin, particularly α1, and highlighting that the NTD is most likely responsible for such binding, as the CTD is partially unfolded.

As for a detailed sidechain analysis, sadly AWSEM is not suitable to answer such questions due to its granularity, but we believe that our data points to said conclusions. Nevertheless, as the presented data is not conclusive, and furthermore we do not possess the experimental results to support this, we have clarified in all instances found that this is a hypothesis based on the results.

9. Fig. 3 and S3: The labeling “+NTD” and “-NTD” is misleading; it should be “full length RfaH” and “isolated CTD” or “NTD-CTD” and “CTD”

Corrected as indicated in the new Fig 4 and Fig S7.

For better comparability the orientation of the CTD should be the same in A and B

Corrected.

Termini should be labeled

Corrected.

The NTD should have a weaker color (e.g. just grey) in order to emphasize what happens with the CTD

Changed as mentioned.

10. Fig. 4: labeling: it should be “RfaH – mimimally frustrated”, “RfaH – highly frustrated” etc

Corrected as indicated in the new Fig 5.

The authors state that the β-intermediate forms more minimally frustrated contacts in the presence of NTD than in the absence. This seems to be true for the overall number of minimally frustrated contacts and for the region preceding strand β2. However, the authors should discuss the fact that there are more minimally frustrated contacts for the isolated CTD than for RfaH especially in strands β3, β4 and the C-terminus.

We thank the reviewer for this recommendation. We analyzed in more detail the contacts that change between the intermediate and the NTD and added a small paragraph that details these results in p 19. In short, the N-terminal region of the CTD in isolation is interacting with final strands and the C-termini of this domain, but when the NTD is present, the N-terminal region of the CTD interacts with the NTD and leads to the reduction of minimally frustrated interactions observed for the C-terminus of the CTD.

11. p. 11: Is the three-strand intermediate observed for NusG-CTD the same as for RfaH-βCTD?

We modified Fig S6 to include a comparison between the CTD of RfaH and NusG, alongside the RMSD differences between them., and added a small comparison in the main text.

12. p. 11: The authors state that NusG CTD also traverses through a three-strand intermediate during refolding. Is this the same intermediate as for RfaH-CTD? Why is this intermediate not shown in Fig. S3?

We thank the reviewer’s question. As mentioned by the reviewer, we colored the points of a trajectory according to the final state they reach, for RfaH this can be either fully folded (red) or partially folded (cyan), yet for the NusG CTD, they always reach the fully folded state (red), that is why we did not show the intermediate in this figure. Nevertheless, we modified Fig S6 to include the refolding trajectories of RfaH and NusG CTD and compare the intermediate structure, as well as labelling the refolding trajectories to indicate when the simulations reach the intermediate state.

13. p.13, l 1: the term “exists in solution as two-domain protein” is strange as it was also a two-protein if the domains interacted (tightly) -> needs rephrasing (see #5)

Rephrased

14. p. 13, l9-10: is the intermediate observed in the analysis of the energy landscapes (Fig. S2) the same as that observed during refolding simulations?

It is indeed the same. We have included a comparison of these structures in Fig. S6 and also briefly commented about their similarity in p 22.

15. p. 13, l 10-14: more details about the involved NTD residues should be provided. It should be discussed if these residues are available when RfaH is bound to the TEC. If yes, these contacts would stabilize the intermediate, preventing the CTD to proceed to the full beta-state, and the authors should discuss this.

We thank the reviewer’s comments. We calculated NTD-CTD contact maps for the intermediate and αRfaH, observing that one interaction patch is the same for both configurations. However, a second interaction patch is formed mainly by non-native interactions between NTD and CTD. Nevertheless, these NTD residues are solvent-protected when bound to the TEC. We show the NTD interaction patches in Fig 5 and discuss the nature of these contacts in p 19.

16. p. 13, l15-16: the authors conclude that the NTD actively destabilizes the β -barrel; this has however not been directly demonstrated. It as only been shown in the refolding simulations that the intermediate is stabilized when switching from random coil to beta. Moreover, if the β -state is trapped in the folding intermediate due to stabilizing contacts, what is the driving force to leave the intermediate towards the beta or the α-state (especially to beta as the energy of the intermediate and the beta state seem similar according to Fig. S2)

We thank the reviewer’s comment. Respect to the phrase that the NTD actively destabilizes the barrel, as indicated previously, we rephrased it to mean that it draws the equilibrium towards the β-intermediate by stabilizing it, which more accurately reflects our observations.

17. p. 15, l 7-8: The authors hypothesize that the βCTD destabilization by the NTD is the initial step for RfaH refolding into the autoinhibited state. It is not obvious why the NTD actively destabilizes the βCTD. Although there seem to be more minimally frustrated contacts for the intermediate than the bCTD, the b-intermediate seems to be comparable to the β-state energetically (Fig. S2).

This energy difference is such because we selected a temperature near the transition of the beta-state to the β-intermediate, so it becomes clear that both exist. In the new Fig 2, it can be seen that the energy basin for the β-intermediate sits at a higher energy than the β-state at temperatures below the folding temperature for the isolated CTD.

18. Discussion: the discussion about the importance of the intermediate may benefit from a scheme that illustrates the refolding steps suggested by the authors (including the most important structures, i.e. autoinhibited, unfolded, intermediate, activated)

We agree with the reviewer’s point of view. We added a simplified scheme (i.e. without structures) as Fig 1 that is inserted in the introduction.

19. In general all physical and mathematical quantities/variables/constants (such as “T”, “Qdiff”, “j”, “Q0”) in the text and in graphs (e.g. Fig. 1B) must be in italics!

We thank the reviewer for raising this issue, we corrected all instances where we found this to happen.

Minor comments:

p. 3, l 4 a reference should be chosen that reviews the modular structure of NusG proteins, e.g. Werner JMB 2012 or Artsimovitch mbio 2019

We agree with the reviewer and added a newer reference in this point.

p. 5, l 5 and 7: only genes are expressed, not proteins

Indeed, we corrected the error.

p. 8, l 21: the chronological order is not correct as Fig. 2B is mentioned before 2A.

We removed the first reference and added a new line introducing the results.

Table S1: nomenclature of RfaH is not the same as in the text (αRfaH vs. RfaH-α)

We corrected every instance that we found.

p. 11, l22-26: the sentence is very long and hard to understand and should be rephrased (divided in two or more sentences)

The sentence was rephrased for clarity.

P. 12, l. 17 the reference should be #34, not #13

Indeed, we changed it.

p.12, l 15-17: The sentence seems not correct

We reformulated it.

p.13, l 1: ref 5 is not correct and should be replaced by Burmann et al BiochemJ 2011

We agreed and changed it.

p. 16, l 22: “diff” must be subscript

We corrected this.

**Reviewer #2:** This work presents the results of folding simulations of the C-terminal domain of the RfaH transcription factor, either isolated or as part of the full-length protein. This domain is known to undergo a transition from a alpha-helical fold to a beta-barrel fold at different stages of its regulation activity. The work appears to highlight the role of interdomain interactions in RfaH in regulating the transformation from one fold to another. However it seems to me that the delivery of the main message of the work, being buried in several layers of other information, could be improved substantially. I am also concerned with several technical aspects of the work as described below. In my opinion, the work in its present form cannot be recommended for publication.

The objectives of the work are very unclear at a structural level. The key description of the system is given in the second paragraph of the Introduction I think. However, personally, I had a very hard time visualizing the main features of the system. A picture/cartoon/diagram could be very useful here to summarize the system and the process. Perhaps the diagram could also pinpoint the interdomain interactions referenced in the following paragraphs that appear to be main subject of study.

We appreciate the reviewer’s comments. We added a new Fig 1 to summarize the fundamentals of the system and indicate the major findings.

I found that, generally, the primary objectives and results of the work are hard to grasp when buried in long discussions interdispersed with acronyms and jargon. I recommend that these are condensed in a brief sentence. What does this work add to the extensive previous modeling studies of this system?
We appreciate the reviewer’s comments. We extensively tried to simplify as much as we could our manuscript, avoid jargon and acronyms unless required, included a new Fig 1 that aids in summarizing the findings of the article and also formulated more concisely the final paragraph of the introduction.

The presentation of the Methods after the Results does not help the presentation. The Results refer to quantities (Qdiff, Qw, etc.) defined in the Methods whose significance is unclear to reader. Sentences such as "RfaH interdomain contacts were kept as bias for simulating the αRfaH state in the full-length system, while all contacts between the βCTD and NTD in the cryo-EM structure were ignored by increasing the distance in the residue-residue distance matrix beyond the threshold from which a contact is considered to take place (9.5 Å)." is very confusing to the reader if terms such as "bias", "distance matrix", "threshold", are not described, at least qualitatively.

We tried, as much as we could, to best describe all of the terms employed throughout our manuscript. We also extensively modified the methods section in a way that most of these terms precede the results.

The AWSEM model comes out a bit from the blue. It would be useful to discuss early one of why it was adopted and why it is believed to be a suitable model for this system. I could not understand what the "memory" potential is, and what is the significance of "single" or "dual" memories.

We added a new paragraph detailing the advantages of studying such a system with the current model. We also explained in more detail in the methods section what are the dual and single memory systems.

Qdiff page 16, should probably be (q-qB)/(qA-qB) so that Qdiff goes from 0 to 1 as the structure goes from B to A as suggested by the language of the paragraph following the third equation on this page. As defined Qdiff's range in not [0,1].

We thank the reviewer for this correction. We checked the code and the equation is (q-qA)/(qB-qA), given the definition q. We also changed the notation for the definition of q, as this is more similar to what the code actually calculated, albeit the numerical results of Q_diff_ are the same.

In the following equation, what is the meaning of rij raised to NA or NB? NA and NB are not defined but the notation suggests that they are the number of residues of the two reference structures? Or are NA and NB simply labeling the two reference structures? If the latter, why not use simply A and B?

Indeed, this is just to indicate the reference structure from which the distance is calculated. Changed it to A and B.

This equation is also dimensionally ill-defined. The exponents should be dimensionless but the units of distance square in the numerator do not match the units of sigmaij, which is dimensionless.

The expression is a gaussian function, which only serves the purpose of giving a distribution so that the contacts between residues close and far in sequence encounter and stablish the interaction during the simulation.

Page 17. Here and elsewhere, supporting data in the the supplementary information are referenced as results. For example "secondary structure assignments are summarized in Table S1 ..." In my opinion, if they are so important to be referenced in the main text, supporting data should be shown in the main text. Otherwise, references should be omitted and the content of the supplementary information should be summarized elsewhere so as not to confuse the reader of which results are essential to the work and which ones are available for confirmation, if needed.

We agree with the concerns raised by the reviewer, and in the current version of the manuscript we minimized the occurrence of this.

**Reviewer #3:** Galaz-Davison et al. describes computer simulations of RfaH, a protein that undergoes so-called fold switching, i.e., a reversible structural transformation from one fold to another. The focus in this work is on the role of the N-terminal domain (NTD) of RfaH for this transition. Fold switching as a phenomenon is becoming increasingly recognized as an important mechanism in both protein function and evolution and RfaH is one of the most well studied fold switching proteins, both experimentally and theoretically. Depite this, there has been very little attention given to role of the NTD (the part of RfaH left unchanged during fold switching) in previous studies, making the present study timely.

A major strength of this work is the folding “annealing” simulations carried out on RfaH and the related protein NusG. NusG is of interest because it is a homolog of RfaH but does not exhibit fold switching. It is shown that for RfaH the folding of the C terminal domain (CTD) into its beta fold is hampered by energetically favorable interactions with the NTD. However, these interactions are not as strong for NusG. For NusG, folding of the CTD proceeds undisturbed even in the presence of the NTD. This is an important results because it hints at the mechanism of the “reverse” fold switch (i.e. how the betaCTD fold reverts back to its alpha fold, which is neccessary to reset RfaH to its autoinhibited state). Overall, the present study is of significant interest for experimental and theoretical researchers in the field of protein fold switching. The manuscript is generally clear and well written (although see points below), and includes a very nice Discussion section.

With the above in mind, I have a few questions and concerns regarding some of the methodology and analysis. These questions should be addressed before the manuscript can be accepted for publication.

One of my main concerns is with the umbrella sampling strategy that are used to obtain the equilibrium behavior both the complete RfaH protein and the C terminal domain (CTD) in isolation. The AWSEM model (used for all simualtions) includes a term V_memory, which is based on information from experimental structures of RfaH. However, it is not clear in the manuscript how V_memory is applied. Page 16 states generally that V_memory “can be guided to multiple structures, or as used in this work, limited to a single reference structure” but further down on the same page “dual memories” are mentioned in relation to the choice of initial structures. How V_memory is incorporated into to the umbrella sampling simulations should be clarified…

We included a brief description on how the fragment memory potential is applied during the umbrella sampling (p 7).

… Since V_memory is central to the present study, I suggest also including sore more general information about this term, e.g., functional form, free parameters, to inform the reader.

We now described the form of the potential in the methods section (p 7).

In the umbrella sampling simulations, the AWSEM model is combined with a harmonic potential in the parameter Q_diff. Does this mean that there are two different types of biases in these simulations (V_memory and the umbrella bias)?

In a certain way, yes, the simulations are biased to acquire structures compatible with the α- and β-folds of RfaH. And through umbrella we sample those very configurations that otherwise would be too difficult to sample at equilibrium.

Are both types of biases needed to capture fold switching?

Performing the simulations without the fragment memories but with the native contacts of both folds could suffice to correctly fold the protein into each configuration, but as the landscape turns rougher, more sampling would be required to carry out a rigorous analysis. Such sampling could be computationally unfeasible.

It would be interesting to see some simulation results of AWSEM without the umbrella sampling, as a point of comparison. Typically, umbrella sampling is seen merely a trick to enhance sampling of conformational space and should in principle not influence the resulting (equilibrium) behavior of the underlying model. Or should the umbrella weight function be seen here as an additional “effective” bias towards the two folds? It would be useful if this issue could be clarified.

We thank the reviewer’s questions and comments. The AWSEM potential with dual memory provides such a rugged free energy landscape that the spontaneous transition between α- and β-fold could hardly be observed without umbrella sampling. As an example, we performed exploratory dual-memory refolding simulations of RfaH (with the duality only in its CTD), and out of 50 simulations, 43 ended up in misfolded conformations. Of the rest, five achieved the correct αRfaH fold, and two the βRfaH. Some of them ended up in the intermediate configuration, highlighting that the free-energy landscape was being sampled, just not enough to drive the whole system towards their minimized configuration. Moreover, in previous articles from our group (<https://doi.org/10.1371/journal.pcbi.1004379>), we could not observe the transformation of RfaH under equilibrium conditions unless we directly manipulated the strength of the interdomain interactions to achieve efficient sampling of both native states. Thus, the enhanced sampling technique is indeed required for AWSEM to rigorously observe the RfaH transformation.

I also have some concerns with the analysis of the umbrella sampling simulations. It seems somewhat optimistic that the results from a single temperature (please specify which T in the text) can be accurately reweighted to span the temperature range ~500-800 Kelvin using WHAM, especially if the simululations were carried out below the folding temperature. It is possible that the simulations sufficiently well samples the unfolded state, since they span states in between the alpha and beta folds. However, this can not be assumed a priori. Could a few separate simulations at higher temperatures be carried out to confirm the results from the WHAM analysis?

We thank for the reviewer’s comment and question regarding the WHAM analysis and umbrella sampling setup. The initial simulations were performed at 650 K for CTD and 630 K for full-length RfaH. We now performed all umbrella sampling simulations at 650 K and 750 K and analyzed them together using WHAM. These results end up making a clearer definition of the energy basins for the folded states and the transitions observed.

Some clarifications are needed regarding the definition of order parameters Qw, Qdiff and q. i) According to page 17, Qw is calculated by summing over all (N-2)(N-3)/2 residue-residue pairs j>i+2. But on page 6, it is mentioned that beta CTD-NTD contacts are ignored by increasing the distance in the residue-residue distance matrix beyond 9.5 Å.

Indeed, we organized all this information in the methods section, so it becomes clearer.

ii) There appears to be a sign error in way Qdiff is defined. Inserting q_A for q leads to Qdiff = 0 and inserting q_B for q gives Qdiff=-1, but the range should presumably be 0 to 1.

Indeed, we corrected the definition of *Q_diff_* based on what is on the code (p 8).

iii) Is it possible to define q in terms of Qw, thereby simplifying expression for q?

Sadly no, although the definition is mathematically similar, the ranges of both equations are different, as *Q_w_* is evaluated for every contact below the threshold in a reference structure, *q* is evaluated for every pair of residues that forms a contact below a threshold in any of the two reference structures. So, numerically the sums of the left and right in the q function will always give values that are slightly lower than *Q_w_A* and *Q_w_B*.

iv) The width of the gaussian functions is not constant but taken to be sigma = |i-j|^0.15? Please comment on this choice.

This is to favor the interaction between residues that are too far apart in sequence separation, as the greater the separation the wider the resulting distribution, and is using such distribution that the developers of the AWSEM force field calibrated this model.

Minor points:

1) How was the value of lambda_FM=0.30 determined? Presumably, this parameter controls the strength of the fragment memory term (please clarity). Why does a higher lambda_FM lead to a stronger cooperativity? These points should be clarified.

We added the expression for V_memory in the main text for clarity (p 7). This value of *λ* is determined simply by visually comparing short temperature unfolding traces at 0.2 and 0.3 values of *λ_memory_* and observing the number of transitions involved in the unfolding process. This value is merely empirical as in our initial refolding attempts, we noticed more than one transition for αRfaH.

2) Page 5 line 21. “Just before” should presumably be “just below”?

Exactly, we corrected it.

3) Page 7, line 7. The free energy surface in Fig. 1C is described as having a “single, deep energy minimum”. I suggest rephrasing this sentence as any protein energy landscape will have a multitude of minor minima throughout its energy landscape.

We agree and rephrased the sentence.

4) Page 16. It would be helpful if the basic idea of umbrella sampling is explained in a few sentences at the beginning of the section “Qdiff and umbrella sampling”.

We added a paragraph describing the use of the method in the methods section, which now precedes the results section (p7).

5) The histograms in Fig. S1 indicate appropriate overlaps between consecutive Qdiff and energy distributions, however, they are hard to read. Perhaps it would be worth trying lines rather than box histograms.

We agree and changed the histograms from Fig S1 (now Fig S2) to lines with filled area.

7) Fig. S4. Refolding “curves” is probably not a good description of these scatter plots.

Good point. We changed them to “refolding plots”.

8) It would be nice to see some actual (re)folding trajectories, showing, e.g., Qw or RMSD values as a function of simulation time.

We agree and included refolding trajectories for every model studied, the isolated CTD of NusG and RfaH are shown in Fig S6, while βRfaH (both folded and intermediate), the RfaH-NusG chimera and αRfaH are displayed in Fig S5.

9) Please include figures showing the full residue-residue distance matrix referenced on page 6. Alternatively, if Qw, q, etc, are determined using sets of native contacts, please report all contact maps, number of contacts in each set, and the criterion for a contact.

We acknowledge the reviewer’s concerns. Nevertheless, we used a distance matrix rather than a contact map (with threshold cutoff), making it inconvenient to directly paste this matrix. We described in the text the criteria for considering a contact in *Q_W_* and *Q_diff_*, and added in Fig S1 a heatmap plot of the distance matrices.

**Reviewer #4:** One of the domains of the protein RfaH folds to a β-rich fold in isolation but switches to an α-rich fold in the presence of its other domain. RfaH has been used as a model protein to understand the molecular basis of fold switching. In this manuscript, the authors simulate RfaH in order to understand the mechanism of fold switching and the effect of the non-switching domain on this mechanism.

Comments:

(1) Since RfaH has been simulated with many models (including the authors’ previous work), it would be useful to have a detailed discussion up front of what each of the models include/exclude in terms of force field and what is gained from using a specific model. This should help to focus on the benefits of using AWSEM as against other models.

We thank the reviewer’s suggestion and added a paragraph describing and comparing AWSEM to our previous approach (p5).

(2) The AWSEM force field has several terms and additionally can be used with or without native structure bias. For readers who have not seen AWSEM before, it would be useful to discuss each of the terms, clearly specifying which of the terms have a native-bias in each of the simulations. This is important for fully grasping the results.

We thank the reviewer’s concern about the terms of the potential. We increased the description of each potential used in AWSEM and mentioned that the fragment memory is the only native bias used (p6 onwards).

(3) It would be useful to have the same tics and tic labels on Figs. 1C and 1D for ease of visual comparison. Is the third minimum (12,16)

Corrected it

in Fig. 1D the unfolded ensemble? What is at (5,5) in 1C and why is it absent in 1D? And where would the beta-barrel intermediate lie on these landscapes?

We now marked the energy minima that we could observe, as well as added in Fig. 1C more details about the basins visited during the transition. Since we now incorporated the data of the higher temperature and extended the equilibration time for the simulations (time that is not considered for the analysis) we no longer observe such features.

The different basins on this figure should be labelled.

We corrected this

The landscapes should be re-labelled free-energy landscapes and free-energy and energy should not be used interchangeably.

We relabeled the landscapes and changed our use of free energy vs energy.

The temperature units should be explained or reduced units should be used since the units are far from real temperatures.

Temperatures on AWSEM have been normalized for only a set of a few proteins. As a result, artificially high temperatures have been observed in the folding process, as has been reported by multiple authors (Kapil et al. 2018, *Phys Chem Chem Phys*; Chen, Zheng & Wolynes. 2016, *PNAS*; Chen et al. 2016, *JACS*). Therefore, we normalized the temperatures using as a reference the peak in heat capacity for αRfaH, and now all the temperatures are reported as fraction of the folding temperature.

(4) The authors state: “One disadvantage of the umbrella sampling simulations is that, by directly employing the number of native contacts of the system in αRfaH and βRfaH as collective variables to drive the structural interconversion of RfaH, the formation or disruption of interdomain contacts between specific residue pairs is also biased.” on page 8. Why does unbiasing not work for this?

The reason we discuss this is because the interdomain native contacts specific a unique conformation, but an unbiased system (i.e. a system in which native contacts are not used for sampling the configuration space) might explore many other binding modes based on sequence information alone. Since we hypothesized that, despite the myriad of possible orientations that could stabilize αCTD when bound to the hydrophobic patch of the NTD, the sequence information within RfaH is sufficient to correctly orient and position the CTD, these simulations had to be run in the absence of these native contact biases.

What else is affected by the umbrella sampling which cannot be unbiased?

We think that sampling the interdomain interface in the multiple configurations that it can take is the only thing that cannot be correctly sampled from the umbrella sampling experiment.

(5) The authors perform a frustratometer analysis of the intermediate with full length RfaH. However, they do not indicate if the extra minimally frustrated contacts that they see are native contacts seen at the αCTD-NTD interface or are these non-native contacts.

We modified Fig 4 (now Fig 5) to include the interaction points between the NTD and CTD, and its comparison with the autoinhibited state. This is described in the main text (p 17 onwards)

Also, if these contacts are native-like then are they between the hydrophobic patch which is present in the NTD of RfaH and absent in NusG.

NusG also displays a less hydrophobic patch that could in principle interrupt RfaH. However, as shown in Fig 5, most of the interactions between NTD and the CTD intermediate are non-native. On the other hand, the observation that the β-barrel of NusG always reaches the native state in our simulation chimera, despite having a strongly hydrophobic patch nearby, suggests that non-metamorphic NusG members exhibit a stable β-barrel that lacks NTD-CTD interactions that could impede its function.

There should be sequence signatures present in the simulations and the analysis performed here which should allow the authors to predict one or a few mutations which destabilize the fold switching or change its kinetics.

We thank the reviewer’s comments, we analyzed the contacts that arise from the interaction between the intermediate and the NTD and show these results in Fig. 5. Furthermore, we added a small paragraph in p 19 detailing the contacts established between the two domains.
