# Supplementary material for: The N-terminal domain of RfaH plays an active role in protein fold-switching
Source: PLoS Comput Biol. 2021 Sep 3;17(9):e1008882. doi: 10.1371/journal.pcbi.1008882 (PMC8454952; doi:10.1371/journal.pcbi.1008882)
Supplement: Attachment — Submitted filename: Reviewer_Responses_rev2.docx [file pcbi.1008882.s010.docx]

**Reviewer's Responses to Questions**

We would first like to thank the reviewers for their positive comments and suggestions to further improve our manuscript. In the following paragraphs, the author’s responses to each comment raised by the reviewers are written in red.

**Reviewer #2:**

The revised methods section is substantially improved relative to the original manuscript. However, at least two of the equations (the equations are not numbered in the manuscript) are still problematic.

Thanks for the suggestion of numbering the equations.

Terms with dimension of distance squared cannot be present as exponents. In my opinion, the manuscript cannot be published unless these are corrected.

The reviewer is correct, the equation has the distance units in the divisor, in our case, in Å, to cancel the distance squared in the numerator. The corrected equation can be found in Sirovetz, Schafer and Wolynes (2017) *Proteins* *85*(11), 2127-2142.

**Reviewer #3:**

The authors have clarified basically all the issues that I raised in my comments, including the functional form of V_memory and the structural parameters (q, Qdiff, etc). Regarding the WHAM analysis, it would have been nice to see a comparison of separate analyses of the two sets of simulations carried out (the results should in principle agree). However, it appears that the WHAM analysis of the combined simulations as presented in the revised manuscript (Fig. 2) are in qualitatively in agreement with those of the original submission, indicating agreement.

From my perspective, the manuscript can be accepted for publication if the following (minor) points are addressed:

1) The description of the umbrella sampling simulations in Methods should be updated to include information about the two different temperatures used.

We thank the reviewer for this suggestion. We included a description of the temperatures used and their conversion to the relative folding temperatures in page 9.

2) It would help the reader if Tf is explained in Results upon first usage in this section.

We briefly combined the description of the temperatures used for the simulations with the explanation of Tf in page 9.

3) Expressing temperatures in units of Tf is probably a good idea. However, I suggest reporting also the nominal value of Tf in AWSEM units, such that the two scales can be linked. This will be helpful in case someone would like to perform similar types of simulations in the future.

We reported the AWSEM temperature units in the same section indicated before. The simulation data is also available in the Open Science Foundation website indicated in the Data Availability section (page 21).

**Reviewer #4:**

The manuscript is mostly okay in its current form. I have three minor comments:

1) Is the memory of the entire RfaH-NTD-alpha-CTD structure encoded in any of the simulations (in particular the refolding simulations)? Or are there only memories of the NTD and the two conformations of the CTD separately? Specifically, are the interdomain interactions between the RfaH NTD and RfaH CTD purely due to the transferable parts of the AWSEM potential? This should be clarified in the manuscript. A similar clarification should be given for the hybrid NusG NTD-RfaH-CTD model.

We thank the reviewer for this question. The simulations do not include a memory for the whole protein, but of each domain separately, such that interdomain interactions are a result of residue-residue interactions and not of fragment memories guiding the domain association process. We indicated this in page 13, line 17:

“By not providing information about contacts between the NTD and CTD, these simulations freely explore the interdomain interaction landscape”.

Nevertheless, based on the question raised by the reviewer, we included another sentence to make this point more explicit on page 14:

“Therefore, any interdomain interaction formed in these simulations is the result of stabilizing residue-residue contacts encoded by the transferable part of the AWSEM force field, and not due to fragment memory or any other external potentials to favor its exploration.”

2) To me, this fold switch looks similar to most ligand induced conformational transitions: the beta-RfaH-CTD is the “open” structure because it has a larger number of stabilizing contacts than alpha-RfaH-CTD. Ligand binding, here binding to the NTD, increases the stabilization of the alpha-RfaH-CTD and this allows the conformational change. If the authors agree, they should put this fold switch in the context of ligand induced conformational transitions in general.

This is a very interesting point about the fold-switch of RfaH in the context of other conformational transitions. However, there are many reasons that have pushed us away from presenting its behavior in such general context.

On one hand, αRfaH is the most stable form of the full-length protein in solution. On the other, the α-to-β transition is triggered by a ligand, which in this case corresponds to the ops-stalled RNA polymerase. Thus, contextualization of the NTD as a ligand for the β-to-α transition of the CTD might mislead other non-expert readers.

A third and relevant element is that RfaH, and many other metamorphic proteins, involved a structural change from one ordered fold topology to a vastly different one. In contrast, conformational changes in non-metamorphic proteins involve “shearing” or “hinge” behavior where entire protein subunits or secondary structure elements undergo relative motions without significantly altering the fold of the protein (10.1016/j.bpj.2020.07.034).

3) The manuscript needs to be copyedited to remove some non-standard phrasing and sentence construction.

We asked one of our long-standing collaborators, who is a native English speaker, to read our manuscript. Based on this feedback, we tried to correct as many non-standard sentences as possible throughout the manuscript.
